# Wilson’s Disease: A Comprehensive Review of the Molecular Mechanisms

**DOI:** 10.3390/ijms16036419

**Published:** 2015-03-20

**Authors:** Fei Wu, Jing Wang, Chunwen Pu, Liang Qiao, Chunmeng Jiang

**Affiliations:** 1 Department of imaging, the Affiliated Zhongshan Hospital of Dalian University, 6 Jiefang Street, Zhongshan District, Dalian 116001, Liaoning, China; E-Mail: wufei0348@126.com; 2 Department of Internal Medicine, the Second Hospital of Dalian Medical University, 467 Zhongshan Road, Shahekou District, Dalian 116023, Liaoning, China; E-Mail: wangjing1224920@sina.com; 3 Department of Biobank, the Sixth People’s Hospital of Dalian, 269 Luganghuibai Road, Ganjingzi District, Dalian 116031, Liaoning, China; E-Mail: dahaixiaowen@sina.com; 4 Storr Liver Centre, Westmead Millennium Institute for Medical Research, Faculty of Medicine, the University of Sydney at Westmead Hospital, Westmead, NSW 2145, Australia

**Keywords:** Wilson’s disease, *ATP7B* gene, copper metabolism, molecular mechanism

## Abstract

Wilson’s disease (WD), also known as hepatolenticular degeneration, is an autosomal recessive inherited disorder resulting from abnormal copper metabolism. Reduced copper excretion causes an excessive deposition of the copper in many organs such as the liver, central nervous system (CNS), cornea, kidney, joints, and cardiac muscle where the physiological functions of the affected organs are impaired. The underlying molecular mechanisms for WD have been extensively studied. It is now believed that a defect in P-type adenosine triphosphatase (*ATP7B*), the gene encoding the copper transporting P-type ATPase, is responsible for hepatic copper accumulation. Deposited copper in the liver produces toxic effects via modulating several molecular pathways. WD can be a lethal disease if left untreated. A better understanding of the molecular mechanisms causing the aberrant copper deposition and organ damage is the key to developing effective management approaches.

## 1. Introduction

Wilson’s disease (WD), also known as hepatolenticular degeneration, is an autosomal recessive disorder resulting from abnormal copper metabolism, subsequently leading to the accumulative deposition of copper in the target organs and impairing the normal functions of the affected organs. WD is not a new disease, first described by Wilson in 1912 [1], but the exact molecular mechanisms leading to the abnormal copper metabolism are a myth. Liver is the major organ for copper deposition in patients with WD. Excessive copper deposition in the liver causes hepatic dysfunction, resulting in a large spectrum of manifestations ranging from mild abnormalities in liver function tests, to acute or chronic hepatitis, cirrhosis, or even fulminant hepatitis [2]. Excessive deposition of the copper in brain may cause neurological disorders such as Parkinson-like symptoms, including bradykinesia, tremor and dystonia, or neuropsychiatric symptoms, such as hypomnesia, dysgnosia, and personality abnormalities [3,4]. The Kayser-Fleischer (KF) ring, a rusty brown ring around the cornea of the eye, is the result of copper deposition in the cornea. Although the KF ring is a significant sign of WD, it is not entirely specific for diagnosis. Copper deposition in other organs may cause corresponding clinical disorders such as osteoarthritis, abnormal kidney function tests, and cardiomyopathy [5].

*ATP7B* is currently believed to be the key culprit gene for WD. Expression of *ATP7B* is found in most organs with a particularly high level in liver, kidney and placenta [6]. *ATP7B* encodes the copper transporting P-type ATPase, a very important enzyme for copper transport in the body. Physiologically, ATP7B plays double roles in liver: It participates in copper transporting to the *trans*-Golgi network (TGN), where copper is incorporated into ceruloplasmin and participates the biliary excretion of copper. Mutation of the *ATP7B* gene is closely linked to the impairment of copper excretion, leading to abnormal deposition of copper in the target organs [7]. Increased tissue copper level may induce a series of harmful biochemical reactions, particularly oxidative stress, which can damage the structure and integrity of mitochondria, leading to cell injury [8,9]. In the clinical setting, a variety of manifestations in patients with WD may be present; Even in patients with the same gene mutation, the clinical manifestation may be heterogeneous. Apart from the mutation of *ATP7B* gene, other factors such as additional genetic modification, lipid metabolism, and even the environmental factors may contribute to the development of WD.

WD is lethal if without timely diagnosis and treatment [10]. The estimated prevalence of WD worldwide is between 1/30,000 and 1/100,000 individuals, and the carrier rate is about 1 in 90 of the population [11]. Early detection and early intervention is critical in preventing the disease progression and irreversible sequalae. A better understanding of the molecular mechanism behind the development of the disease is imperative. In this review, we aim to briefly summarize the recent development of the molecular mechanisms involved in the development of WD.

## 2. The Molecular Architecture of P-Type ATPase (ATP7B)

The currently available data suggest that *ATP7B* belongs to P-type ATPase family gene and is located on the chromosome 13q.14.3 [12]. P-type ATPase (ATP7B) is a critical enzyme essential for copper transport. *ATP7B* consists of 20 introns and 21 exons. Several domains of this gene contribute to copper transport. The *N*-terminal metal-binding domains (MBDs) comprise of six heavy metal-associated sites, each of which contains the repetitive sequence motif GMXCXXC. Antioxidant protein 1 (Atox1), one of the copper chaperones, has been shown to help deliver copper to ATP7B [13]. The MBDs have a high affinity for copper and play a major role in the acceptance of copper from Atox1 by special protein–protein interactions [14]. It has been thought that the ancillary role of Atox1 in selectively delivering copper to MBD2 is an essential step for further copper migration [15]. However, it was recently suggested that MBDs are not an indispensable domain for copper transport, since mutation of the MBDs alone perturbs but does not completely suppress the trafficking of ATP7B [16,17]. Mutation analysis has proven that MBD5 and MBD6 can influence the catalysis and activation of ATP7B activity, but MBD1-4 has less impact on ATP7B [18].

Previous studies have shown that a putative transmembrane channel consisting of eight discontinuous ion channels may contribute to copper transport [19]. The Cys–Pro–Cys (CPC) sequence motif located in the sixth transmembrane domain is highly conserved across all ATPases. CPC is thought to be a part of the intramembrane metal-binding site and may promote copper transportation; however, it is not essential for copper-induced trafficking of ATP7B [20,21].

Transportation of copper is dependent on the energy provided by ATP hydrolysis. The characteristic feature of all ATP7B is that they all contain an ATP-binding domain (also called nucleotide-binding domain, or N-domain), a phosphorylation domain (P-domain), and a phosphatase domain (A-domain) [17]. The N-domain was determined with bond ATP and participates in the domain–domain interactions [22]. There is a unique amino acid motif (histidine is an invariant residue) that is highly conserved in the N-domain [23]. Although it is not clear how the SEHPL motif influences the copper transport, correlation between the mutation of SEHPL motif and WD has been identified [14]. The mutation of this motif (His1069Q) represents the most common mutation of WD in northern European populations [23]. The P-domain contains a highly conserved sequence motif DKTGT which is critical for enzyme phosphorylation [24]. Phosphorylation of the aspartic acid residue (Asp) from the sequence DKTGT is required for copper transportation. The acyl-phosphate is the phosphorylated intermediate that can drive the protein conformation changes and the copper transport to the opposite side of the membrane [25]. The A-domain is the place where the acyl-phosphate gets dephosphorylated. A-domain contains a Thr–Gly–Glu sequence motif (TGE) in which the Glu residue is required for the phosphatase to function. Dephosphorylation of acyl-phosphate marks the completion of the cycle [26].

Although the *C*-terminal is dispensable for catalysis, it plays an important role in the maintenance of protein stability and the regulation of protein location [20,27].

**Figure 1:**
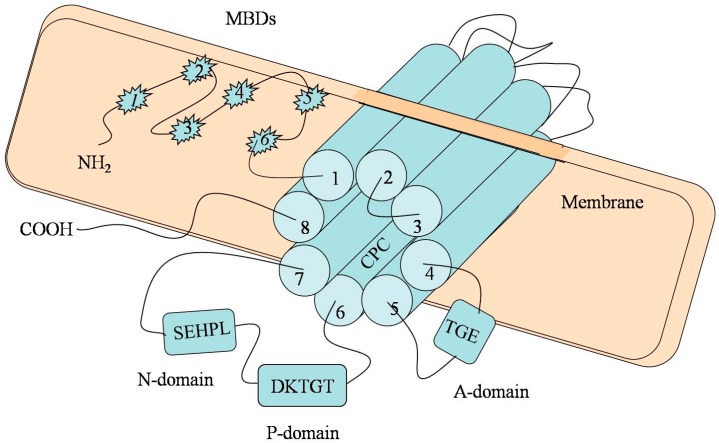
Diagram of *ATP7B*. The metal-binding domain contains six copper-binding domains (MBD1-6), all with the conserved sequence motif CXXC. The transmembrane channel consisting of eight discontinuous ion channels may contribute to copper transport (Cylinder1-8). The Cys–Pro–Cys (CPC) sequence motif is the key residue that confers metal ion selectively. ATP binds to the N-domain and the SEHPL motif located in the N-domain. The P-domain is the room for phosphorylation of Asp from the sequence DKTGT. The A-domain is the place where the acyl-phosphate gets dephosphorylated. Mutations may occur at any position of the gene, and then may cause the deposition of copper.

A schematic illustration of the structure of *ATP7B* is presented in Figure 1.

## 3. Function and Regulation of ATP7B in Copper Transportation

ATP7B is essential for copper distribution and excretion in the human body. The gene *ATP7B* is expressed in most organs, and is particularly rich in liver. Intracellularly, ATP7B is mainly located in the TGN [28,29]. Binding of the copper to ATP7B provokes ATP hydrolysis, producing energy for copper transport from the cytosol to Golgi lumen [30]. More recent studies have indicated that ATP7B is redistributed from TGN to the late endosome/lysosome with the increase of copper concentration in hepatocytes [31,32].

**Figure 2:**
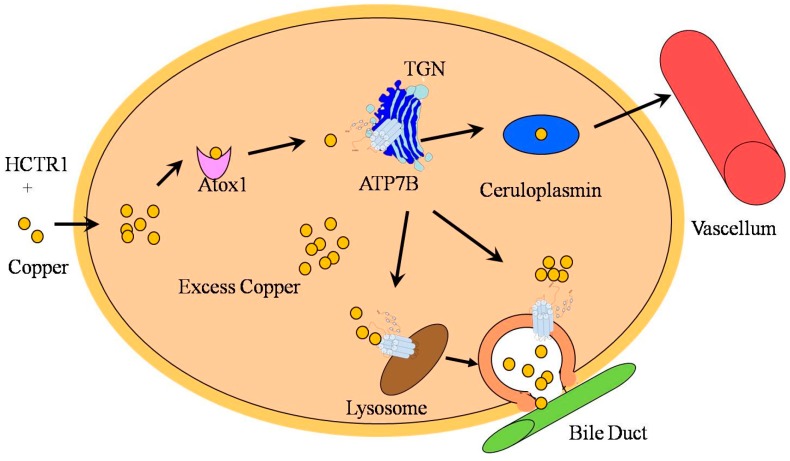
Copper is delivered by HCTR1 to cytosol where it mainly binds to Atox1. Atox1 transfers copper to TGN and is transported into the lumen with the help of ATP7B. Copper is then incorporated into ceruloplasmin which is then released to vascellum. The excess copper facilitates ATP7B trafficking from the TGN to the lysosome, then copper can be transported to the lysosomal lumen, and the excess copper is excreted to bile via exocytosis. An increasing level of copper also stimulates ATP7B to move to cytosolic vesicles where copper is isolated and then released into the bile duct.

ATP7B plays a double role in liver: It participates in copper transportation to TGN where copper is incorporated into ceruloplasmin, and it is involved in the biliary excretion of copper. Copper is mainly absorbed in the intestine by ATP7A (a member of the P-type ATPase family, it shares a similar structure and function with ATP7B and is mainly expressed in the intestinal epithelium.) [33], and is delivered to the hepatocyte by HCTR1 (a high-affinity copper transport protein) [34]. After entry into the hepatocyte, copper is distributed to different intracellular compartments such as mitochondria, nucleus, and cytosol. Binding of copper to Atox1 in cytosol and shifting to TGN where copper is incorporated into ceruloplasmin with the help of ATP7B is among the important processes of copper transport [35]. Excess copper facilitates ATP7B trafficking from the TGN to the lysosome, leading to the copper being transported to the lysosomal lumen. Once the level of copper reaches a threshold value, excess copper is excreted to bile via exocytosis [36]. On the other hand, increased copper level can also simulate ATP7B to move into cytosolic vesicles where copper may be “isolated”, hence the cells are protected from the copper induced toxicity. Copper in vesicles is thought to be excreted by a similar mechanism as in the lysosomes (exocytosis) [37]. A schematic illustration of copper transport and metabolism is presented in Figure 2.

## 4. Mutation Hotspots of *ATP7B*

Worldwide, nearly 500 mutations in *ATP7B* have been identified. Among these, more than 300 mutations have been found to be associated with WD [38]. Mutations may occur at any position of the gene, including exons, introns and even promoter regions. The most common form of *ATP7B* mutation is missense mutation. Other forms include frameshift mutation, nonsense mutation, and splicing mutation. Generally, frameshift and missense mutation are associated with a more severe phenotype of WD [39].

It has long been noted that a genetic heterogeneity in patients with WD exists across various races and geographical regions [40]. For example, H1069Q mutation of the *ATP7B* is more prevalent in WD patients of European origin such as those from Italy, Romania, and Sweden [41], whereas the R778L mutation is the more common in East Asia [42]. None of these two mutations have been found in India where 17 other mutations have been identified [43]. In theory, a consistent mutation spectrum would be useful in making the pre-symptomatic diagnosis for WD patients; however, due to the large variability in *ATP7B* mutation and gene heterozygocity, it is difficult to utilize the mutation signatures as a diagnostic tool [44].

## 5. Mechanism in Copper-Induced Liver Injury

Aberrantly increased intracellular levels of copper have been shown to induce oxidative stress through the Fenton reaction [45]. Excess copper in cells can stimulate the production of hydroxyl radicals which could damage the lipids, protein, and nucleic acids [46]. Mitochondria are the major targets for oxidative damage resulting from copper toxicity. In particular, copper-induced dysregulation of lipid has been shown to significantly contribute to mitochondrial injury [47]. Cardiolipin (CL) is the main lipid component of the mitochondrial membrane, and it plays an important role in maintaining the integrity and function of the mitochondrial membrane [48]. Reactive oxygen species (ROS) and lipid peroxidation induce the fragmentation of CL and thus regulate the mitochondrial permeability and cell death [49,50]. The phosphatidic acid (PA) and phosphatidyl hydroxyl acetone (PHA) were demonstrated to be the major products of fragmental CL [51,52]. Although a direct causal relationship between the copper toxicity and the formation of PA or PHA is lacking, studies by high-performance thin-layer chromatography and MALDI-TOF-MS (Matrix-Assisted Laser Desorption/Ionization Time of Flight Mass Spectrometry) have revealed an increased level of PA and PHA in the liver mitochondria of *ATP7b^−/−^* mice but not the wild-type control mice [53]. Recent studies have shown that PA not only plays a role in phospholipid biosynthesis, but is also an important lipid messenger. PA can be recognized by many proteins, and they can regulate multiple cellular processes such as cell transformation, cytoskeletal organization, cell proliferation, survival, and tumor progression [54]. Copper-induced CL fragmentation impairs the homeostasis of PA. It has been shown that fragmentation of CL destroys the integrity and function of mitochondria and leading to cytochrome c release, thereby initiating apoptosis pathway and cell injury [55].

Recent studies indicate that the Fenton-like reaction happens in the late phase of WD [56,57]. In a cell-free system, the characteristic changes of mitochondrial ultrastuctural damage such as enlargement of intermembrane space and condensations within the intracellular organelles were observed at the initial phase of WD under the electron microscopy. These changes, considered to be the consequence of the multivalent interactions or multiprotein crosslinking, are believed to be reversible by copper-chelating therapies [56].

Despite these reported data, the precise mechanisms by which excess copper causes tissue injury are still not entirely clear, and thus warrant further studies.

## 6. Modifying Factors and Phenotypic Diversity in WD

In a previous study involving two pairs of monozygotic (MZ) twins with WD who were *ATP7B* H1069Q homozygous, it was shown that their clinical manifestations were different [58], suggesting that in addition to the allelic heterogeneity, there must be other factors contributing to the phenotypic diversity in patients with WD [59,60]. In a recent study conducted in a mountainous population with a high prevalence of WD, the *ATP7B* gene in seven patients and their 43 family members were sequenced and the genotype and phenotype correlation was evaluated [61]. It was revealed that the onset of disease, predominant manifestations, and time of diagnosis were similar among the individuals tested, indicating there might be an environmental factor involved in the disease phenotypes. Evidently, the genotype–phenotype relationship in the patients with WD is multifactorial [62,63,64], and it is difficult to identify a direct genotype–phenotype correlation in all patients with WD.

Epigenetic differences, in addition to the allelic heterogeneity, may also contribute to the phenotypic diversity in patients with WD. DNA methylation is one of the basic regulations of gene expression. *S*-adenosylmethionine (SAM) is the methyl donor for DNA methylation. DNA methylation is achieved under the catalysis of DNA methyltransferase (DNMTs). The *S*-adenosylhomocysteine (SAH) is the product as well as inhibitor of the transmethylation [65], and the *S*-adenosylhomocysteinase (AHCY) is the hydrolase for SAH. In a Jackson toxic milk mouse (tx-j) model of WD, the AHCY transcript levels were negatively correlated with the accumulation of cooper [66]. Accumulation of copper restrains the hydrolysis of SAH and leads to an increased intracellular level of SAH, in turn inhibiting the level of DNMT transcripts [67], reduced transmethylation [68] and subsequent alteration in gene expression. A significant difference in the expression level of DNMTs had been seen between healthy livers and livers of the patients with chronic hepatitis, cirrhosis and hepatocellular carcinomas [69].

Hepatic steatosis is a common feature of patients with WD. Previous studies have shown that an increased intracellular copper level could inhibit the lipid synthesis, particularly synthesis for cholesterol [70]. An animal model of WD was performed to analyze the metabolism of cholesterol in *Atp7b^−/−^* mice, in which a marked down-regulation in the synthesis of cholesterol was observed. However, the target gene of sterol regulatory-binding protein 2 (SREBP-2), a transcription factor which activates cholesterol biosynthesis, also showed a significant down-regulation [71]. It was speculated that the accumulation of copper inhibits the function of SREBP-2 [71].

Overall, the relationship between genotype and phenotype in patients with WD is still not clearly defined, and further studies are warranted.

## 7. Conclusions

Wilson’s disease is an inherited metabolic disease and is lethal if left without timely diagnosis and treatment. Due to the heterogeneity of the clinical manifestations, misdiagnosis is common, thereby leaving many patients without timely treatment. Thus, early detection of WD is a critical component of proper management. Unfortunately, an effective early diagnostic procedure for WD is lacking.

Based on previous experience, the diagnostic work-up should include the following: careful history taking, clinical manifestation, laboratory examinations such as liver function tests, serum ceruloplasmin, 24 h urinary excretion of copper, ophthalmologic examination, liver biopsy, measurement of the hepatic parenchymal copper level, hepatic imaging studies, and genetic testing [72]. The “gold standard” of diagnosis for WD is still liver biopsy [73]. With the increased disease awareness and advancement of medical genetics, genetic testing in the affected patients and their family members, as well as in individuals suffering from unexplained liver or neuropsychiatric diseases, will be playing a more important role [74,75]. However, a widespread screening for culprit gene mutation can be very costly and is thus less practically useful for all populations [76].

The current treatment options for patients with WD include medical therapy and liver transplantation. Copper chelator is the first-line and effective treatment option for WD, despite some adverse effects [77]. Zinc has been recommended as a maintenance drug for the treatment of WD as it helps prevent copper absorption from intestinal tract [78].

Liver transplantation is the treatment of choice for patients with WD who have fulminant hepatic failure or the end stage of cirrhosis [79]. Even if the liver function is stable, liver transplantation should still be considered as a definitive therapy for WD in patients who have crippling neurological diseases [80,81]. Over recent years, human-induced pluripotent stem cells (iPSC) have provided a unique platform for the treatment of WD [82], as the “man-made hepatocytes” generated by iPSC technology could be used as functional surrogate cells for the “sick” hepatocytes [83]. In addition, human embryonic stem cells and fetal liver stem cells may all hold potential as the source cells for the treatment of WD [84].

## Abbreviations

ATP7B: P-type adenosine triphosphatase
CL: Cardiolipin
DNMTs: DNA methyltransferase
MBDs: Metal-binding domains
PA: Phosphatidic acid
SAH: *S*-adenosylhomocysteine
SAM: *S*-adenosylmethionine
TGN: *Trans*-Golgi network
WD: Wilson disease
